# Bis(acetato-κ*O*)bis­(thio­urea-κ*S*)cobalt(II)

**DOI:** 10.1107/S1600536814002074

**Published:** 2014-02-05

**Authors:** Martin Lutz

**Affiliations:** a Bijvoet Center for Biomolecular Research, Crystal and Structural Chemistry, Faculty of Science, Utrecht University, Padualaan 8, 3584 CH Utrecht, The Netherlands

## Abstract

The title compound, [Co(CH_3_COO)_2_(CH_4_N_2_S)_2_], is isotypic with the corresponding Zn^II^ complex. The metal atom is in a distorted tetra­hedral coordination environment with the two S atoms from two thio­urea ligands and two O atoms from two acetate anions as the coordinating atoms. All H atoms of the thio­urea ligands are involved in N—H⋯O and N—H⋯S hydrogen bonds, leading to a three-dimensional network.

## Related literature   

For the isotypic Zn^II^ compound, see: Cavalca *et al.* (1967 ▶). For a definition of tetra­hedral distortion, see: Robinson *et al.* (1971 ▶).
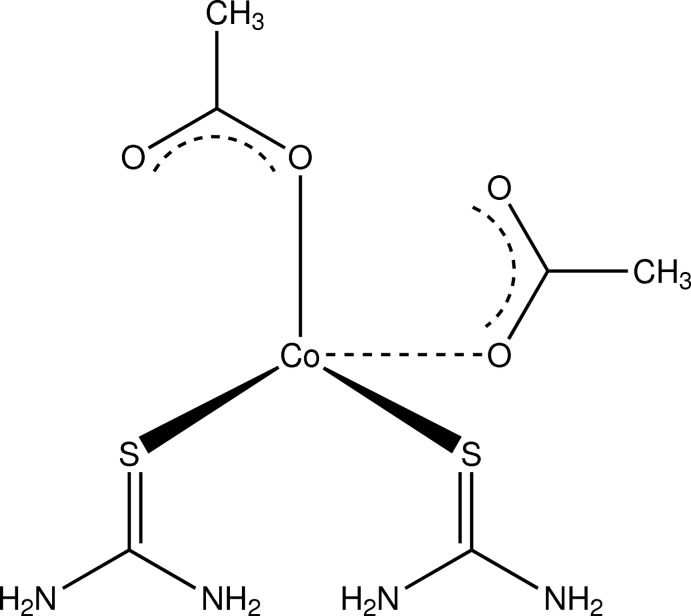


## Experimental   

### 

#### Crystal data  

[Co(C_2_H_3_O_2_)_2_(CH_4_N_2_S)_2_]
*M* *_r_* = 329.26
Monoclinic, 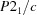

*a* = 7.15257 (16) Å
*b* = 17.2864 (4) Å
*c* = 11.7372 (3) Å
β = 112.275 (1)°
*V* = 1342.92 (5) Å^3^
*Z* = 4
Mo *K*α radiation
μ = 1.60 mm^−1^
*T* = 150 K
0.26 × 0.15 × 0.10 mm

#### Data collection  

Bruker Kappa APEXII diffractometer
Absorption correction: numerical (*SADABS*; Sheldrick, 2012 ▶) *T*_min_ = 0.730, *T*_max_ = 0.871
27333 measured reflections
3087 independent reflections
2898 reflections with *I* > 2σ(*I*)
*R*_int_ = 0.018

#### Refinement  

*R*[*F* ^2^ > 2σ(*F* ^2^)] = 0.016
*wR*(*F* ^2^) = 0.041
*S* = 1.04
3087 reflections
190 parameters
H atoms treated by a mixture of independent and constrained refinement
Δρ_max_ = 0.35 e Å^−3^
Δρ_min_ = −0.19 e Å^−3^

### 

Data collection: *APEX2* (Bruker, 2007 ▶); cell refinement: *Peakref* (Schreurs, 2013 ▶); data reduction: *Eval15* (Schreurs *et al.*, 2010 ▶) and *SADABS* (Sheldrick, 2012 ▶); program(s) used to solve structure: initial coordinates from the literature (Cavalca *et al.*, 1967 ▶); program(s) used to refine structure: *SHELXL2013* (Sheldrick, 2008 ▶); molecular graphics: *PLATON* (Spek, 2009 ▶); software used to prepare material for publication: *SHELXL2013*.

## Supplementary Material

Crystal structure: contains datablock(s) I, global. DOI: 10.1107/S1600536814002074/sj5387sup1.cif

Structure factors: contains datablock(s) I. DOI: 10.1107/S1600536814002074/sj5387Isup2.hkl

CCDC reference:

Additional supporting information:  crystallographic information; 3D view; checkCIF report

## Figures and Tables

**Table:** 

Co1—O3	1.9462 (8)
Co1—O1	1.9847 (8)
Co1—S1	2.3291 (3)
Co1—S2	2.3299 (3)

**Table:** 

O3—Co1—O1	101.57 (3)
O3—Co1—S1	112.22 (3)
O1—Co1—S1	95.07 (2)
O3—Co1—S2	117.69 (3)
O1—Co1—S2	117.47 (3)
S1—Co1—S2	110.445 (11)

**Table 2:** Hydrogen-bond geometry (Å, °)

*D*—H⋯*A*	*D*—H	H⋯*A*	*D*⋯*A*	*D*—H⋯*A*
N1—H1⋯O4^i^	0.830 (18)	1.959 (18)	2.7717 (14)	165.8 (16)
N1—H2⋯O2^ii^	0.877 (16)	1.959 (17)	2.8324 (14)	173.6 (14)
N2—H3⋯S1^iii^	0.881 (19)	2.859 (19)	3.7200 (12)	165.7 (15)
N2—H4⋯S2^i^	0.825 (16)	2.810 (16)	3.5080 (11)	143.5 (14)
N2—H4⋯O4^i^	0.825 (16)	2.613 (17)	3.2479 (15)	134.8 (14)
N3—H5⋯O1^iv^	0.810 (16)	2.482 (16)	3.1759 (13)	144.5 (14)
N3—H6⋯O2	0.810 (17)	2.038 (17)	2.8388 (14)	169.6 (16)
N4—H7⋯O1^iv^	0.848 (16)	2.108 (17)	2.8994 (14)	155.3 (14)
N4—H8⋯O3^v^	0.808 (17)	2.176 (16)	2.8452 (13)	140.3 (14)

Symmetry codes: (i) 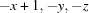; (ii) 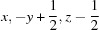; (iii) 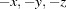; (iv) 
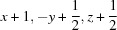
; (v) 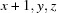.

**Fig. 1.**
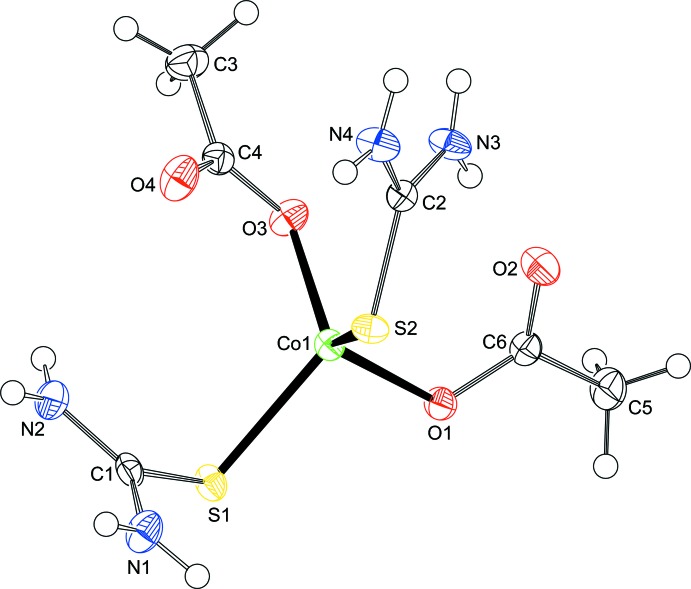
: Molecular structure of the title compound. Displacement ellipsoids are drawn at the 50% probability level. H atoms are drawn as small spheres of arbitrary radii. Only the major conformations of the rotationally disordered methyl groups are shown.

**Fig. 2.**
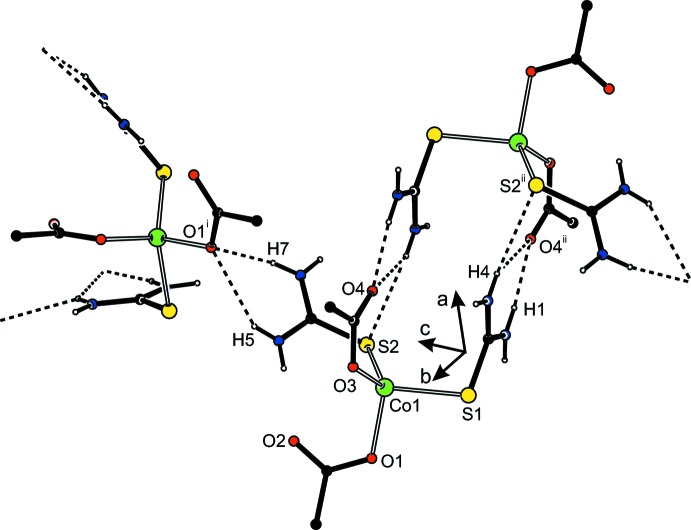
: Hydrogen bonds involving the bifurcated acceptor atoms O1 and O4, and the bifurcated donor atom H4. Overall, the crystal structure consists of a three-dimensional hydrogen bonded network. Methyl H atoms are omitted in the drawing. Symmetry codes: (i) x + 1, 1/2 - y, z + 1/2; (ii) 1 - x, -y, -z.

## supplementary crystallographic information

### 1. Comment

The crystal structure of bisthiourea-zinc acetate has been described in the literature in the centrosymmetric space group *P*2_1_/*c* (Cavalca *et al.*, 1967). The corresponding cobalt compound was mentioned to be isotypic but no coordinates or further structural information were given. We therefore set out to crystallize the title compound and to determine its crystal structure.

It could indeed be confirmed that the title compound is isotypic with the zinc complex from the literature. The coordinates of the Zn compound were used as starting model for the least-squares refinement of the present Co structure. The metal center is in a distorted tetrahedral environment with two S atoms from two thiourea ligands and two O atoms from two acetate molecules as coordinating atoms (Figure 1). Coordination angles between 95.07 (2) and 117.69 (3) ° lead to an angle variance (Robinson *et al.*, 1971) of 81.93 °^2^. The two Co—S distances are equal within standard uncertainties and are, as expected, longer than the Co—O distances. With a difference of 0.0385 (11) Å, the Co1—O1 distance is significantly longer than the Co1—O3 distance. A possible explanation for this difference are the hydrogen bonding interactions (Table 2). O1 the is acceptor of two hydrogen bonds, while O3 is the acceptor of only one.

A comparison of the Co environment of the present study with the Zn environment from the literature (Cavalca *et al.*, 1967) remains inconclusive because of the large standard uncertainties of the Zn structure, which had been obtained at room temperature from film data. The difference in the two metal-S distances described for the Zn complex could not be detected in the Co complex (see Table).

The quality of the present low-temperature study allowed a detailed analysis of the H atoms. In the difference-Fourier maps, the two methyl groups of the acetate ligands appeared to be orientationally disordered. In the refinement, an idealized disorder model was used with a 60 ° rotation between the disorder forms. This disorder model was allowed to rotate about the C—C bond, and the H atom occupancies were refined. In the case of C3, the major disorder form has an occupancy of 0.881 (17) and is eclipsed with respect to the carboxylate [H3A—C3—C4—O4 - 9 °]. The major component at C5 has an occupancy of 0.626 (17)% and is in *gauche* conformation to the carboxylate [H5A—C5—C6—O1 - 32 °]. In the crystal packing, the methyl groups are surrounded only by other methyl groups.

All H atoms of the thiourea ligands are donors of hydrogen bonds (Table 2). O1 and O4 are bifurcated acceptors of hydrogen bonds, and H4 is a bifurcated hydrogen bond donor (Figure 2). The angle sum at H4 is 358 (2) °. The intermolecular hydrogen bonds involving H5 and H7 as donors and O1 as acceptor result in a one-dimensional chain in the [201] direction. Together with the hydrogen bonds of H2 in the [001] and H8 in the [100] direction, a two-dimensional hydrogen bonded network is formed in the *a*,*c* plane. These two-dimensional sheets are linked in the *b* direction *via* centrosymmetric ring-type hydrogen bonds involving H1 and H4 (graph set *R*_2_^2^(16), symmetry code 1 - *x*, -*y*, -*z*), and H3 (graph set *R*_2_^2^(8), symmetry code -*x*, -*y*, -*z*). H6 is involved in an intramolecular hydrogen bond with O2 as acceptor. Overall this is a hydrogen bonded three-dimensional network.

### 2. Experimental

0.23 g Cobalt(II) acetate tetrahydrate (0.92 mmol) and 0.14 g thiourea (1.84 mmol) were dissolved in deionized water and slowly evaporated at room temperature. Colourless needle-shaped crystals of thiourea and blue block-shaped crystals of the title compound were obtained.

### 3. Refinement

The methyl groups were refined with a model of perfect disorder using the *SHELXL* instruction AFIX 127. The occupancies of the disorder components were refined and the sum of the occupancies was constrained to 1. The H atoms of the thiourea ligands were refined freely with isotropic displacement parameters.

### Crystal data

**Table:**

[Co(C_2_H_3_O_2_)_2_(CH_4_N_2_S)_2_]	*F*(000) = 676
*M**_r_* = 329.26	*D*_x_ = 1.629 Mg m^−^^3^
Monoclinic, *P*2_1_/*c*	Mo *K*α radiation, λ = 0.71073 Å
*a* = 7.15257 (16) Å	Cell parameters from 23812 reflections
*b* = 17.2864 (4) Å	θ = 1.9–27.5°
*c* = 11.7372 (3) Å	µ = 1.60 mm^−^^1^
β = 112.275 (1)°	*T* = 150 K
*V* = 1342.92 (5) Å^3^	Block, blue
*Z* = 4	0.26 × 0.15 × 0.10 mm

### Data collection

**Table:**

Bruker Kappa APEXII diffractometer	2898 reflections with *I* > 2σ(*I*)
Radiation source: sealed tube	*R*_int_ = 0.018
φ and ω scans	θ_max_ = 27.5°, θ_min_ = 2.2°
Absorption correction: numerical (*SADABS*; Sheldrick, 2012)	*h* = −9→9
*T*_min_ = 0.730, *T*_max_ = 0.871	*k* = −22→22
27333 measured reflections	*l* = −15→15
3087 independent reflections	

### Refinement

**Table:**

Refinement on *F*^2^	Primary atom site location: heavy-atom method
Least-squares matrix: full	Secondary atom site location: difference Fourier map
*R*[*F*^2^ > 2σ(*F*^2^)] = 0.016	Hydrogen site location: difference Fourier map
*wR*(*F*^2^) = 0.041	H atoms treated by a mixture of independent and constrained refinement
*S* = 1.04	*w* = 1/[σ^2^(*F*_o_^2^) + (0.0204*P*)^2^ + 0.4848*P*] where *P* = (*F*_o_^2^ + 2*F*_c_^2^)/3
3087 reflections	(Δ/σ)_max_ < 0.001
190 parameters	Δρ_max_ = 0.35 e Å^−^^3^
0 restraints	Δρ_min_ = −0.19 e Å^−^^3^

### Special details

**Table:**

Geometry. All e.s.d.'s (except the e.s.d. in the dihedral angle between two l.s. planes) are estimated using the full covariance matrix. The cell e.s.d.'s are taken into account individually in the estimation of e.s.d.'s in distances, angles and torsion angles; correlations between e.s.d.'s in cell parameters are only used when they are defined by crystal symmetry. An approximate (isotropic) treatment of cell e.s.d.'s is used for estimating e.s.d.'s involving l.s. planes.

### Fractional atomic coordinates and isotropic or equivalent isotropic displacement parameters (Å^2^)

**Table:**

	*x*	*y*	*z*	*U*_iso_*/*U*_eq_	Occ. (<1)
Co1	0.17413 (2)	0.17567 (2)	0.01182 (2)	0.01481 (5)	
S1	−0.00290 (4)	0.08869 (2)	−0.14310 (3)	0.01872 (7)	
S2	0.48512 (4)	0.20539 (2)	0.00160 (2)	0.01697 (6)	
O1	−0.03808 (12)	0.25675 (5)	−0.04056 (7)	0.01876 (16)	
O2	0.16498 (12)	0.33000 (5)	0.11096 (8)	0.02399 (18)	
O3	0.17158 (12)	0.14576 (5)	0.17108 (8)	0.02089 (17)	
O4	0.42393 (13)	0.06561 (5)	0.19563 (9)	0.02632 (19)	
N1	0.26745 (16)	0.03991 (6)	−0.23051 (10)	0.0241 (2)	
H1	0.362 (3)	0.0120 (10)	−0.2305 (15)	0.037 (4)*	
H2	0.226 (2)	0.0787 (9)	−0.2822 (15)	0.029 (4)*	
N2	0.25861 (17)	−0.02779 (6)	−0.06544 (10)	0.0235 (2)	
H3	0.207 (3)	−0.0340 (10)	−0.0085 (16)	0.042 (5)*	
H4	0.351 (2)	−0.0564 (9)	−0.0652 (14)	0.030 (4)*	
N3	0.55520 (16)	0.26384 (6)	0.22516 (10)	0.0212 (2)	
H5	0.626 (2)	0.2701 (9)	0.2971 (15)	0.026 (4)*	
H6	0.439 (3)	0.2784 (9)	0.1970 (15)	0.030 (4)*	
N4	0.82560 (15)	0.21118 (7)	0.19799 (10)	0.0224 (2)	
H7	0.898 (2)	0.2240 (9)	0.2711 (15)	0.027 (4)*	
H8	0.877 (2)	0.1869 (9)	0.1586 (15)	0.027 (4)*	
C1	0.19179 (16)	0.02918 (6)	−0.14601 (10)	0.0174 (2)	
C2	0.63192 (16)	0.22861 (6)	0.15317 (10)	0.0155 (2)	
C3	0.2882 (2)	0.07120 (8)	0.35350 (11)	0.0268 (3)	
H3A	0.3761	0.0265	0.3871	0.040*	0.881 (17)
H3B	0.1487	0.0576	0.3409	0.040*	0.881 (17)
H3C	0.3328	0.1146	0.4113	0.040*	0.881 (17)
H3D	0.1956	0.1060	0.3724	0.040*	0.119 (17)
H3E	0.4231	0.0748	0.4187	0.040*	0.119 (17)
H3F	0.2390	0.0179	0.3483	0.040*	0.119 (17)
C4	0.29873 (16)	0.09407 (6)	0.23235 (10)	0.0169 (2)	
C5	−0.16131 (19)	0.37960 (8)	−0.01005 (12)	0.0276 (3)	
H5A	−0.2394	0.3777	−0.0990	0.041*	0.626 (17)
H5B	−0.1001	0.4309	0.0125	0.041*	0.626 (17)
H5C	−0.2509	0.3695	0.0340	0.041*	0.626 (17)
H5D	−0.1542	0.4077	0.0640	0.041*	0.374 (17)
H5E	−0.2935	0.3545	−0.0475	0.041*	0.374 (17)
H5F	−0.1427	0.4159	−0.0690	0.041*	0.374 (17)
C6	0.00216 (17)	0.31923 (6)	0.02420 (10)	0.0172 (2)	

### Atomic displacement parameters (Å^2^)

**Table:**

	*U*^11^	*U*^22^	*U*^33^	*U*^12^	*U*^13^	*U*^23^
Co1	0.01240 (8)	0.01554 (8)	0.01686 (8)	0.00185 (5)	0.00595 (6)	−0.00089 (5)
S1	0.01406 (12)	0.01684 (13)	0.02354 (14)	0.00173 (10)	0.00521 (10)	−0.00399 (10)
S2	0.01458 (12)	0.02337 (14)	0.01401 (12)	−0.00060 (10)	0.00661 (10)	−0.00146 (10)
O1	0.0178 (4)	0.0179 (4)	0.0189 (4)	0.0044 (3)	0.0050 (3)	−0.0015 (3)
O2	0.0168 (4)	0.0255 (4)	0.0263 (4)	0.0029 (3)	0.0044 (3)	−0.0068 (3)
O3	0.0172 (4)	0.0251 (4)	0.0222 (4)	0.0067 (3)	0.0094 (3)	0.0051 (3)
O4	0.0249 (4)	0.0251 (4)	0.0342 (5)	0.0095 (4)	0.0171 (4)	0.0058 (4)
N1	0.0229 (5)	0.0224 (5)	0.0298 (6)	0.0090 (4)	0.0131 (4)	0.0048 (4)
N2	0.0241 (5)	0.0192 (5)	0.0259 (5)	0.0065 (4)	0.0079 (4)	0.0020 (4)
N3	0.0149 (5)	0.0302 (5)	0.0172 (5)	0.0031 (4)	0.0047 (4)	−0.0068 (4)
N4	0.0148 (5)	0.0321 (6)	0.0200 (5)	0.0034 (4)	0.0063 (4)	−0.0052 (4)
C1	0.0146 (5)	0.0130 (5)	0.0211 (5)	−0.0013 (4)	0.0028 (4)	−0.0049 (4)
C2	0.0148 (5)	0.0162 (5)	0.0164 (5)	−0.0005 (4)	0.0068 (4)	0.0004 (4)
C3	0.0283 (6)	0.0313 (7)	0.0209 (6)	−0.0026 (5)	0.0094 (5)	0.0038 (5)
C4	0.0142 (5)	0.0171 (5)	0.0184 (5)	−0.0018 (4)	0.0049 (4)	−0.0009 (4)
C5	0.0242 (6)	0.0221 (6)	0.0324 (7)	0.0096 (5)	0.0060 (5)	−0.0012 (5)
C6	0.0171 (5)	0.0174 (5)	0.0192 (5)	0.0027 (4)	0.0091 (4)	0.0009 (4)

### Geometric parameters (Å, º)

**Table:**

Co1—O3	1.9462 (8)	N3—H6	0.810 (17)
Co1—O1	1.9847 (8)	N4—C2	1.3169 (14)
Co1—S1	2.3291 (3)	N4—H7	0.848 (16)
Co1—S2	2.3299 (3)	N4—H8	0.808 (17)
S1—C1	1.7420 (11)	C3—C4	1.5051 (16)
S2—C2	1.7356 (11)	C3—H3A	0.9800
O1—C6	1.2889 (14)	C3—H3B	0.9800
O2—C6	1.2365 (14)	C3—H3C	0.9800
O3—C4	1.2833 (14)	C3—H3D	0.9800
O4—C4	1.2338 (14)	C3—H3E	0.9800
N1—C1	1.3107 (16)	C3—H3F	0.9800
N1—H1	0.830 (18)	C5—C6	1.5039 (15)
N1—H2	0.877 (16)	C5—H5A	0.9800
N2—C1	1.3227 (15)	C5—H5B	0.9800
N2—H3	0.881 (19)	C5—H5C	0.9800
N2—H4	0.825 (16)	C5—H5D	0.9800
N3—C2	1.3181 (14)	C5—H5E	0.9800
N3—H5	0.810 (16)	C5—H5F	0.9800
			
O3—Co1—O1	101.57 (3)	C4—C3—H3E	109.5
O3—Co1—S1	112.22 (3)	H3A—C3—H3E	56.3
O1—Co1—S1	95.07 (2)	H3B—C3—H3E	141.1
O3—Co1—S2	117.69 (3)	H3C—C3—H3E	56.3
O1—Co1—S2	117.47 (3)	H3D—C3—H3E	109.5
S1—Co1—S2	110.445 (11)	C4—C3—H3F	109.5
C1—S1—Co1	101.21 (4)	H3A—C3—H3F	56.3
C2—S2—Co1	102.52 (4)	H3B—C3—H3F	56.3
C6—O1—Co1	115.82 (7)	H3C—C3—H3F	141.1
C4—O3—Co1	117.42 (7)	H3D—C3—H3F	109.5
C1—N1—H1	119.2 (12)	H3E—C3—H3F	109.5
C1—N1—H2	120.3 (10)	O4—C4—O3	122.41 (11)
H1—N1—H2	120.2 (15)	O4—C4—C3	121.89 (11)
C1—N2—H3	119.4 (11)	O3—C4—C3	115.70 (10)
C1—N2—H4	121.4 (11)	C6—C5—H5A	109.5
H3—N2—H4	119.2 (16)	C6—C5—H5B	109.5
C2—N3—H5	119.0 (11)	H5A—C5—H5B	109.5
C2—N3—H6	119.7 (11)	C6—C5—H5C	109.5
H5—N3—H6	121.3 (15)	H5A—C5—H5C	109.5
C2—N4—H7	119.6 (10)	H5B—C5—H5C	109.5
C2—N4—H8	121.8 (11)	C6—C5—H5D	109.5
H7—N4—H8	118.5 (15)	H5A—C5—H5D	141.1
N1—C1—N2	120.36 (11)	H5B—C5—H5D	56.3
N1—C1—S1	119.58 (9)	H5C—C5—H5D	56.3
N2—C1—S1	120.03 (9)	C6—C5—H5E	109.5
N4—C2—N3	118.69 (11)	H5A—C5—H5E	56.3
N4—C2—S2	119.51 (9)	H5B—C5—H5E	141.1
N3—C2—S2	121.79 (9)	H5C—C5—H5E	56.3
C4—C3—H3A	109.5	H5D—C5—H5E	109.5
C4—C3—H3B	109.5	C6—C5—H5F	109.5
H3A—C3—H3B	109.5	H5A—C5—H5F	56.3
C4—C3—H3C	109.5	H5B—C5—H5F	56.3
H3A—C3—H3C	109.5	H5C—C5—H5F	141.1
H3B—C3—H3C	109.5	H5D—C5—H5F	109.5
C4—C3—H3D	109.5	H5E—C5—H5F	109.5
H3A—C3—H3D	141.1	O2—C6—O1	122.82 (10)
H3B—C3—H3D	56.3	O2—C6—C5	120.65 (10)
H3C—C3—H3D	56.3	O1—C6—C5	116.53 (10)
			
Co1—S1—C1—N1	−99.80 (9)	Co1—O3—C4—O4	2.53 (15)
Co1—S1—C1—N2	82.07 (9)	Co1—O3—C4—C3	−178.52 (8)
Co1—S2—C2—N4	145.78 (9)	Co1—O1—C6—O2	2.57 (14)
Co1—S2—C2—N3	−34.62 (10)	Co1—O1—C6—C5	−176.89 (8)

### Hydrogen-bond geometry (Å, º)

**Table:**

*D*—H···*A*	*D*—H	H···*A*	*D*···*A*	*D*—H···*A*
N1—H1···O4^i^	0.830 (18)	1.959 (18)	2.7717 (14)	165.8 (16)
N1—H2···O2^ii^	0.877 (16)	1.959 (17)	2.8324 (14)	173.6 (14)
N2—H3···S1^iii^	0.881 (19)	2.859 (19)	3.7200 (12)	165.7 (15)
N2—H4···S2^i^	0.825 (16)	2.810 (16)	3.5080 (11)	143.5 (14)
N2—H4···O4^i^	0.825 (16)	2.613 (17)	3.2479 (15)	134.8 (14)
N3—H5···O1^iv^	0.810 (16)	2.482 (16)	3.1759 (13)	144.5 (14)
N3—H6···O2	0.810 (17)	2.038 (17)	2.8388 (14)	169.6 (16)
N4—H7···O1^iv^	0.848 (16)	2.108 (17)	2.8994 (14)	155.3 (14)
N4—H8···O3^v^	0.808 (17)	2.176 (16)	2.8452 (13)	140.3 (14)

Symmetry codes: (i) −*x*+1, −*y*, −*z*; (ii) *x*, −*y*+1/2, *z*−1/2; (iii) −*x*, −*y*, −*z*; (iv) *x*+1, −*y*+1/2, *z*+1/2; (v) *x*+1, *y*, *z*.

### Comparison of the coordination environment of the Co complex of the present study with the isotypic Zn complex from the literature (Cavalca *et al.*, 1967).

**Table:**

	M=Co	M=Zn	Δ [Å]
M-S1	2.3291 (3)	2.326 (2)	0.003 (2)
M-S2	2.3299 (3)	2.261 (4)	0.069 (4)
M-O1	1.9847 (8)	1.973 (6)	0.012 (6)
M-O3	1.9462 (8)	1.954 (8)	-0.008 (8)
